# Expression of a specific variant surface glycoprotein has a major impact on suramin sensitivity and endocytosis in *Trypanosoma brucei*

**DOI:** 10.1096/fba.2019-00033

**Published:** 2019-09-30

**Authors:** Natalie Wiedemar, Michaela Zwyer, Martin Zoltner, Monica Cal, Mark C. Field, Pascal Mäser

**Affiliations:** ^1^ Swiss Tropical and Public Health Institute Basel Switzerland; ^2^ University of Basel Basel Switzerland; ^3^ School of Life Sciences University of Dundee Dundee United Kingdom

**Keywords:** drug resistance, human African trypanosomiasis, low‐density lipoprotein, sleeping sickness, VSG

## Abstract

Suramin was introduced into the clinic a century ago and is still used to treat the first stage of acute human sleeping sickness. Due to its size and sixfold negative charge, uptake is mediated through endocytosis and the suramin receptor in trypanosomes is thought to be the invariant surface glycoprotein 75 (ISG75). Nevertheless, we recently identified a variant surface glycoprotein (VSG^Sur^) that confers strong in vitro resistance to suramin in a *Trypanosoma brucei rhodesiense* line. In this study, we introduced *VSG^Sur^* into the active bloodstream expression site of a *T. b. brucei* line. This caused suramin resistance and cross resistance to trypan blue. We quantified the endocytosis of different substrates by flow cytometry and showed that the expression of VSG^Sur^ strongly impairs the uptake of low‐density lipoprotein (LDL) and transferrin, both imported by receptor‐mediated endocytosis. However, bulk endocytosis and endocytosis of the trypanolytic factor were not affected, and the *VSG^Sur^*‐expressors did not exhibit a growth phenotype in the absence of suramin. Knockdown of ISG75 was synergistic with *VSG^Sur^* expression, indicating that these two proteins are mediating distinct suramin resistance pathways. In conclusion, VSG^Sur^ causes suramin resistance in *T. brucei* bloodstream forms by decreasing specific, receptor‐mediated endocytosis pathways.

**Abbreviations**
ISG75: invariant surface glycoprotein 75
LDL: low‐density lipoprotein
VSG: variant surface glycoprotein

## 1 INTRODUCTION

Sleeping sickness, transmitted by the tsetse fly, is caused by two subspecies of the protozoan parasite *Trypanosoma brucei*: *T. b. gambiense* typically establishes a chronic infection, whereas *T. b. rhodesiense* causes a rather acute form of the disease. A third subspecies, *T. b. brucei*, is not infective to humans but, together with other trypanosome species, causes livestock trypanosomiasis. Trypanosomes are protected from the mammalian immune system by multiple mechanisms that confound both innate and acquired defenses. The surface coat consists of a dense layer of a single variant surface glycoprotein (VSG); with a reservoir of more than 2000 *VSG* genes and pseudogenes,^1^ trypanosomes evade their mammalian host's adaptive immune responses by antigenic variation. Thus there is no vaccine for sleeping sickness and chemotherapy relies on few available drugs, although more are entering the treatment pipeline.^2^ Suramin is the oldest drug but remains used for the treatment of the first stage of *T. b. rhodesiense* infection.

Despite the use of suramin for a hundred years, its mode of action against African trypanosomes is not completely understood. Suramin inhibits a number of different enzymes including many in the glycolytic pathway.^3^ As a negatively charged molecule, suramin cannot cross membranes by diffusion and was proposed to be taken up through receptor‐mediated endocytosis.^4^, ^5^ More recent RNAi target sequencing studies showed that knock‐down of a number of endosomal and lysosomal genes leads to up to about 11‐fold suramin resistance,^6^ substantiating an uptake route through endocytosis. Invariant surface glycoprotein 75 (ISG75) was proposed to act as the suramin receptor, since its knock‐down led to a decrease in suramin sensitivity.^6^ Suramin shows a high plasma protein binding with approximately 70% bound to albumin, 15% to low‐density lipoprotein (LDL) and 15% free.^5^ Based on findings that trypanosomes took up >20‐fold more suramin in the presence of LDL than in the presence of plasma, whereas the binding and uptake of LDL were up to 70% reduced in the presence of high micromolar concentrations of suramin,^5^ suramin was proposed to be taken up in complex with LDL. However, procyclic trypanosomes overexpressing the trypanosome ortholog of Rab4 showed a 50% decrease in suramin binding and almost no suramin uptake when compared with parental cells, whereas the uptake of LDL was unchanged.^7^ The expression of a constitutively activated version of Rab5 led to increased LDL uptake without affecting suramin uptake, indicating that uptake of LDL and suramin are likely independent.^7^

While resistance to suramin is widespread in the animal pathogen *Trypanosoma evansi* with 50% inhibitory concentrations (IC_50_) of up to 40 µg/mL,^8^, ^9^ there have not been any reports of suramin resistance in *T. brucei* spp. from the field. Nevertheless, resistance can be selected for under in vitro and in vivo conditions in the laboratory.^10^ We recently described a variant surface glycoprotein, VSG^Sur^, which is linked to suramin resistance.^11^ VSG^Sur^ was identified by forward genetics. We had exposed *T. b. rhodesiense* STIB900 clones to high suramin concentrations (80‐fold the IC_50_), and within only one week they became resistant. Transcriptome sequencing showed that all the resistant lines had switched to express *VSG^Sur^*. This phenomenon was reproducible in multiple independent experiments. The *VSG^Sur^* expressing parasites showed an increase in IC_50_ of almost 100‐fold for suramin, and cross resistance to trypan blue, a trypanocidal dye related to suramin. We quantified the amount of intracellular trypan blue and observed a reduced uptake by *VSG^Sur^* expressing parasites.^11^ In this study, we investigate the effects of enforced *VSG^Sur^* expression in a suramin‐sensitive *T. b. brucei* line, aiming to uncover the molecular mechanisms underlying the link between *VSG^Sur^* expression and suramin resistance.

## 2 MATERIALS AND METHODS

Chemicals were purchased from Merck KGaA, Darmstadt, Germany, if not otherwise stated.

### 2.1 *T. brucei* strains and cell culture

The Lister 427 derived *T. b. brucei* 2T1 strain^12^ was cultivated in Iscove's Modified Dulbecco's culture Medium complemented according to Hirumi^13^ and supplemented with 10% heat‐inactivated fetal calf serum. Cells were cultured at 37°C, 5% CO_2._

### 2.2 Introduction of *VSG^Sur^* into 2T1 cells

The plasmid previously described^11^ was modified for the genomic replacement of the *VSG^221^* by *VSG^Sur^* in 2T1 cells. First the coding sequence of *VSG^900^* within the plasmid was replaced by the coding sequence of *VSG^Sur^* (GenBank accession MF093647), which was derived by PCR from cDNA of STIB900_c1_sur1 with primers containing XbaI and AscI restriction sites (VSGsur_XbaI_F: taatctagaatgcaagccgtaacacgc, VSGsur_AscI_R: aatggcgcgccttaaaaaagcaaaaatgcaagc). The construct's framing UTRs used for homologous recombination were replaced with the UTR‐regions of *VSG^221^*. For the 5′ genomic UTR of *VSG^221^* a PCR product amplified from 2T1 gDNA was cloned into the plasmid. Primer used for the amplification of the 5′ genomic UTR of *VSG^221^* contained HindIII and NcoI sites (VSG221_5′UTR_HindIII_f: tagaagcttcaagcacaatttcatcctcct and VSG221_5′UTR_NcoI_r: catccatgggccgcgttcgtgtcg). Correct cloning was validated by sequencing. The 3′ UTR was replaced during the final PCR on plasmid DNA using a forward primer, which binds to the 221 5′UTR sequence (VSG221_5′UTR_HindIII_f), and a reverse primer, which binds to the stop‐codon of *VSG^Sur^* and contains an overhang of 71 nucleotides from the *VSG^221^* 3′UTR sequence. The resulting PCR product was framed by the 3′ and 5′ UTRs of *VSG^221^* and contained a blasticidine‐resistance gene, an αβ tubulin trans‐splice site and the coding sequence of *VSG^Sur^*. Five micrograms of the purified PCR product were used for the transfection of 4 × 10^7^ 2T1 cells in 100 µL Tb‐BSF buffer^14^ with an Amaxa Nucleofector using program Z‐001. Positive‐transfected clones were obtained by limiting dilution in standard culture medium and after 24 hours of incubation, blasticidine was added to a total concentration of 10 and 20 µg/mL. Four positive‐transfected clones and the untransfected parent strain were analyzed by qPCR regarding the expression of *VSG^Sur^* and *VSG^221^*.

### 2.3 RNA isolation and quantitative PCR

Parasites in the exponential growth phase were washed once with PBS and total RNA was isolated using the RNeasy Mini Kit (Qiagen) including an on‐column DNase treatment. SuperScript III Reverse Transcriptase (Invitrogen via Thermo Fisher Scientific) and an oligo(dT) primer were used to synthesize complementary DNA (cDNA) out of 1‐5 µg total RNA according to the manufacturer's protocol. Quantitative PCR was carried out in a Power SYBR Green Mix (Applied Biosystems via Thermo Fisher Scientific) on a StepOnePlus real‐time PCR machine (Applied Biosystems) in technical duplicates. The primers used for qPCR were tcattcgtttggccatacgc and ttactgacagacgaaccggc for *VSG^Sur^* and aaggccaagaaagcg and ttggtaacgcctgttttg for *VSG^221^*. The amplification plots were analyzed with StepOne Software v2.3. and the Ct values were normalized to the housekeeping gene telomerase reverse transcriptase using the ∆∆Ct method.

### 2.4 Drug and human serum sensitivity assays

Suramin (sodium salt) (S2671) and pentamidine isethionate (P0547) were obtained from Sigma (now Merck), trypan blue (93590) from Fluka (now Merck) and melarsoprol from Sanofi‐Aventis/WHO. In vitro drug sensitivities were determined with Alamar blue assays^15^ using standard growth medium. Serial drug dilutions were prepared on a 96‐well plate and parasites were added to a final concentration of 1 × 10^4^ cells/mL. After incubation for 69 hours, resazurin was added to a final concentration of 11.4 µg/mL. Cells were incubated for 2‐4 more hours and fluorescence of viable cells was quantified using a SpectraMax reader (Molecular Devices) and SoftMax Pro 5.4.5 Software. Dose‐response curves were fitted with a nonlinear regression model (variable slope; four parameters, lowest value set to zero) and IC50 values were calculated with GraphPad Prism 6.00. Human serum sensitivity was determined using Alamar blue assays as above. But, instead of drug addition, limited dilutions were prepared with human serum and the medium used was HMI‐9 supplemented with 15% heat‐inactivated horse serum (i‐horse). Human serum was obtained through centrifugation of whole blood of volunteers, heat inactivated at 56°C and stored at −20°C. Specific killing due to trypanolytic factor was checked by incubation of *T. b. gambiense* in 5% human serum of the same batch. *T. b. gambiense* cells were not lysed.

### 2.5 Trypan blue uptake

Cells were harvested in the logarithmic growth phase and resuspended in standard growth medium at a concentration of 4 × 10^5^ cells/mL. Trypan blue was added to the respective concentrations and cells incubated for 2 hours at 37°C. Subsequently, cells were centrifuged for 5 minutes at 3000 *g* at room temperature and washed twice with PBS. Cells were resuspended in 250 µL PBS and fixed in 5% formalin. Fluorescence intensities of the cells were measured with a BD FACSCalibur™ flow cytometer (BD Biosciences). A total of 10 000 events were acquired within a gate of intact cells based on size versus granularity (FSC/SSC) and fluorescence of the gated cells was measured in the FL3 filter. The measured fluorescence intensities were analyzed with Flowing Software (version 2.5.1) using a refined FSCxSSC gate to exclude cell debris and an additional size versus fluorescence gate to exclude parasites that had already been dead during incubation with trypan blue and thus gave a much stronger signal. Fluorescence values were defined as the geometrical mean fluorescence of gated particles (>6000 per replicate).

### 2.6 ISG75 turnover

For each cell line and each timepoint, 40 mL cell cultures were prepared the day before the experiment. The cell density was adjusted for each sample to reach 1.2 × 10^ ^cells/mL. The next day, at the respective time points, cycloheximide (C7698 Sigma, now Merck) was added to the cultures to a concentration of 100 µg/mL. At timepoint zero, the cell densities were measured and the same number of cells was taken from each replicate. Cells were centrifuged for 10 minutes, 1000 *g* at room temperature. The supernatant was removed and the cell pellet resuspended in 1 mL PBS supplemented with protease inhibitor (cOmplete™, Mini, EDTA‐free Protease Inhibitor Cocktail, Roche: 1 tab in 20ml) and centrifuged for 10 minutes, 1000 *g* at room temperature. The cells were washed one more time with PBS containing protease inhibitor, supernatant was removed and 10 µL of 3 × Laemmli buffer added. Cells were further diluted with Laemmli buffer to reach a density of 5 × 10^6^ cells/ 10 µl. Cells were sonicated and incubated at 70°C for 10 minutes. Samples were centrifuged at full speed in a microcentrifuge for 2 minutes and subsequently 10 µL of sample was loaded on a NuPAGE 4%‐12% Bis‐Tris Protein Gel (Fisher Scientific ‐ UK Ltd) and run for 1 hour at 200 V in NuPAGE™ MOPS SDS Running Buffer (Fisher Scientific ‐ UK Ltd). Separated proteins were transferred to a polyvinylidene difluoride (PVDF) membrane (Immobilon‐P; Millipore) in a TE22 wet transfer tank (GE healthcare) in SDS‐Page running buffer supplemented with 20% methanol. The membrane was blocked with blocking buffer consisting of Tris‐buffered saline with 0.01% Tween‐20 (TBST) and 5% milk powder. Subsequently the membrane was incubated for 1 hour in blocking buffer containing the primary antibody. For ISG75 a 1:2,500 diluted polyclonal rabbit antibody was used and for EF1α a monoclonal mouse antibody (clone CBP‐KK1; Millipore) was used in a 1:20 000 dilution. Membranes were washed with TBST three times for 5 minutes and incubated for one hour with a secondary goat anti‐rabbit peroxidase‐conjugated IgG (A0545, Sigma, now Merck) at a 1:20 000 dilution. Membranes were washed once with TBS and twice with PBS before the addition of 1 mL Amersham ECL Western Blotting Detection Reagent (GE Healthcare Life Sciences). After two minutes of incubation, chemiluminescence was detected with a G:box imager (Syngene). Signals were quantified using ImageJ (Fiji) software.^16^

### 2.7 ISG75 knock‐down

A previously described pRPa plasmid for RNAi‐mediated knockdown of ISG75^6^ was amplified in DH5α cells and digested with XhoI. A total of 6 µg purified digested plasmid was used to transfect 4 × 10^7^ *VSG^221^*, respectively, *VSG^Sur^* expressing 2T1 cells. Transfection was carried out as described above and positive transfectants were selected with 10 µg/mL Hygromycin (Invivogen). Pseudoclones were picked after five days and RNAi‐knockdown was induced by the addition of 1 µg/mL Tetracycline. To quantify the level of ISG75 knock‐down, 72 hours after induction, RNA of induced and non‐induced clones was isolated and qPCR was performed as described above^11^ (Wiedemar et al, 2018).^11^ Primers used for qPCR were ISG75qPCR_F (gcttgggttgcttgtgttct) and ISG75qPCR_R (tcgtatttttgcttttagcattagc).

### 2.8 Suramin wash‐out assay

Serial drug dilutions were prepared on a 96‐well v‐shaped plate with the following media: HMI‐9 with 15% inactivated horse serum, HMI‐9 without serum, HMI‐9 without serum but complemented with 10 µg/mL LDL and HMI‐9 without serum but complemented with 0.5% bovine serum albumin (BSA, A7030). Parasites were washed twice with pre‐warmed IMDM, eluted in the respective media and added to the assay plates to a final volume of 100 µL/well and a concentration of 1 × 10^5^ cells/mL for the HMI‐9 medium with 15% inactivated horse serum. Since the cells in the media without serum were not dividing during the incubation time, higher inocula were used and parasites were added to a concentration of 2 × 10^5^ cells/mL. Plates were incubated for 4 hours at 37°C, 5% CO_2_ and subsequently centrifuged at 1000 *g* for 3 minutes. 50 µL of the supernatant was removed and 150 µL pre‐warmed IMDM was added to all the wells. Cells were washed three more times, each time 150 µL supernatant was removed and 150 µL IMDM or, in case of the last wash, 150 µL HMI‐9 with 15% i‐horse was added. After the last centrifugation 150 µL supernatant was removed and 50 µL HMI‐9 with 15% i‐horse was added to elute the parasites. A new flat‐bottom plate was prepared with 50 µL HMI‐9 with 15% i‐horse and 50 μL of the eluted parasites were transferred to the new plate. The flat‐bottom plate was incubated for another 64 hours before resazurin was added to a final concentration of 11.4 µg/mL. After another 4‐5 hours of incubation at 37°C, 5% CO_2_, fluorescence of viable cells was measured with a SpectraMax (Molecular Devices) and SoftMax Pro 5.4.5 Software. Dose‐response curves were fitted with a nonlinear regression model (variable slope; four parameters, lowest value set to zero) and IC50 values were calculated with GraphPad Prism 6.00.

### 2.9 Uptake and binding of transferrin and low‐density lipoprotein

To quantify the uptake of transferrin and LDL, cells in the logarithmic growth phase were washed once with serum‐free medium supplemented with 1% BSA and resuspended in serum‐free medium supplemented with 1% BSA at a concentration of 2 × 10^6^ cells/mL. 392 µL (for the transferrin uptake experiment) and 95 µL (for the LDL uptake experiment) of resuspended parasites were transferred to Eppendorf tubes. Cells were pre‐incubated for 15 minutes at 37°C to internalize surface bound transferrin and LDL. At the respective timepoints fluorescently labeled transferrin (Alexa Fluor™ 488 Conjugate, T13342, Fisher Scientific, Illkirch Cedex, France) was added to a concentration of 50 µg/mL or fluorescently labeled LDL (Bodipy FL, I34359, Fisher Scientific, Illkirch Cedex, France) to a concentration of 10 µg/mL. At time point zero, cells were quenched by putting them on ice and by the addition of 1 mL ice‐cold PBS. Cells were centrifuged for 5 minutes at 1000 *g* at 4°C and washed once with ice cold PBS. Cells were fixed and fluorescence quantified as described above.

To quantify the impact of suramin on the uptake of transferrin and LDL, cells were washed once with IMDM and eluted in IMDM without BSA to a concentration of 2 × 10^6^ cells/mL. The experiment was performed without BSA, since otherwise the majority of suramin would bind to BSA. Cells were pre‐incubated for 15 minutes (transferrin) or for one hour (LDL) at 37°C. Suramin was added to concentrations between 0 and 1000 µmol/L and subsequently transferrin or LDL were added to concentrations of 5 or 10 µg/mL, respectively. Cells were incubated for 10 minutes (transferrin) or for 20 minutes (LDL) at 37°C, quenched, washed, and fixed as described above.

For the quantification of transferrin and LDL binding to the cell surface, cells were washed with IMDM and eluted in IMDM to a concentration of 1 × 10^7^ cells/mL. Cells were pre‐incubated for 15 minutes (transferrin) or for one hour (LDL) at 37°C and subsequently chilled on ice for 15 minutes. Either suramin or cold PBS was added and subsequently transferrin or LDL were added to a concentration of 800 or 100 µg/mL, respectively. Cells were incubated for 10 minutes on ice, subsequently washed twice with ice‐cold PBS and fixed as described above.

### 2.10 Concanavalin A uptake and binding

Trypanosomes in the logarithmic growth phase were centrifuged at 1000 g for 5 minutes, the cell pellet was eluted in IMDM with 1% bovine serum albumin (BSA) to a concentration of 2 × 10^6^ cells/mL. 498 μL of eluted parasites were put in 1.5 ml Eppendorf tubes and pre‐incubated at 37°C for 15 minutes. At the respective time points FITC conjugated Concanavalin A (C7642 Sigma, now Merck) was added to a concentration of 5 µg/mL. After incubation at 37°C cells were quenched on ice by the addition of ice‐cold PBS. Subsequently cells were centrifuged for 5 minutes at 1000 *g* at 4°C and washed once with PBS. Cells were eluted in 250 µL PBS and fixed by the addition of 250 µL 10% formalin.

For the quantification of surface bound ConA, cells were centrifuged and eluted in IMDM with 1% BSA to a concentration of 2 × 10^6^ cells/mL. These were pre‐incubated on ice for 15 minutes and subsequently the ConA was added to concentrations of 0, 5, 20, and 50 µg/mL. After incubation on ice for 10 minutes, ice‐cold PBS was added, the cells centrifuged at 4°C (5 minutes, 1000 *g*) and subsequently washed one more time with ice‐cold PBS, eluted in 250 µL PBS, and fixed by the addition of 250 µL 10% formalin.

Fluorescent intensities of the cells were measured as described above but using the FL1 filter. Fluorescence values were defined as the geometrical mean fluorescence of >9000 events.

## 3 RESULTS

### 3.1 Expression of *VSG^Sur^* renders *T. b. brucei* resistant to suramin

**Figure 1:**
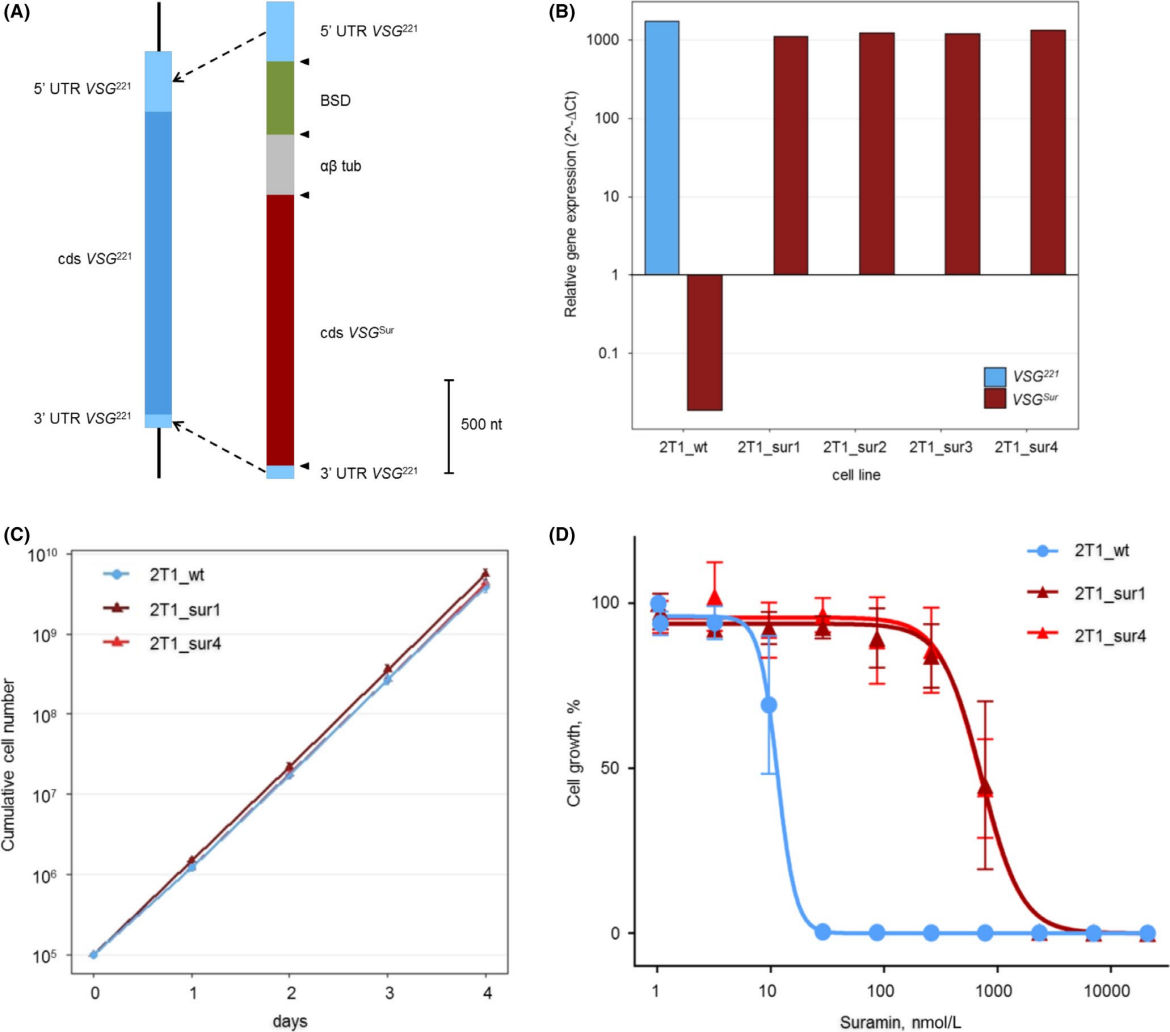
Introduction of *VSG^Sur^* into the active bloodstream expression site renders 2T1 cells suramin resistant. A, Schematic illustration of *VSG^221^* within the active bloodstream expression site of 2T1 cells before transfection and of the construct used for the in situ replacement of *VSG^221^* with *VSG^Sur^*. The construct is framed with the 5′ region of *VSG^221^* with adjacent sequence on one side and the 3′ region of *VSG^221^*on the other side; it contains a blasticidine resistance gene (BSD), an αβ tubulin splice site (αβ tub), and the coding sequence (cds) of *VSG^Sur^*. Triangles indicate restriction‐sites. B, Expression of *VSG^Sur^* and *VSG^221^* as determined by qPCR in the parent 2T1 strain (2T1_wt) and in four transfected clones (2T1_sur1‐4). Values shown are derived from technical duplicates; they represent expression levels normalized to the housekeeping gene *TERT*. The transfected clones show a high expression level of *VSG^Sur^*, comparable to the expression level of *VSG^221^* in the 2T1 parent cells. C, Cumulative cell number as measured over four days, error bars represent standard deviations (n = 3). D, Dose‐growth curves for suramin show an 58‐fold decrease in suramin sensitivity after introduction of *VSG^Sur^* (n = 3)

To test the hypothesis that the expression of *VSG^Sur^* is sufficient to cause suramin resistance in *T. brucei*, we introduced the *VSG^Sur^* gene into the active *VSG* expression site of *T. b. brucei* 2T1, a well‐established *T. b. brucei* bloodstream‐form laboratory line.^12^ 2T1 cells naturally express *VSG^221^*,^17^ also called MITat1.2 (GenBank: X56762.1). To replace *VSG^221^* with *VSG^Sur^* we made a construct (Figure 1A) which contained the coding sequence of *VSG^Sur^* together with a blasticidine resistance gene, framed by the sequences of the 5’ and 3’ regions of *VSG^221^*. Four transfected clones were recovered, all of which showed no expression of *VSG^221^* and expressed *VSG^Sur^* at the same level as the parental line expressed *VSG^221^* (determined by qPCR using telomerase reverse transcriptase (*TERT*) as a reference; Figure 1B). Two of the *VSG^Sur^* expressing transfectants (2T1_sur1 and 2T1_sur4) were chosen for further analysis. Proliferation was essentially indistinguishable from the parent cell line when cultivated in standard medium without blasticidine (Figure 1C). The 50% inhibitory concentration (IC_50_) for suramin increased from 12 nmol/L in the *VSG^221^* expressing parent to 700 nmol/L in the two *VSG^Sur^* expressing transfectants (*P* = 0.0036, One‐Way ANOVA; Figure 1D). No alterations of drug sensitivities were observed for melarsoprol or pentamidine, excluding a non‐specific drug resistance mechanism. However, the *VSG^Sur^* expressing cells showed weak cross resistance to trypan blue, with an elevation of the IC_50_ from 13 µmol/L in the *VSG^221^* expressing parent to 19 and 21 µmol/L in the *VSG^Sur^* expressing transfectants, but this was statistically non‐significant.

### 3.2 *VSG^Sur^* expressing cells show a reduced uptake of trypan blue

**Figure 2:**
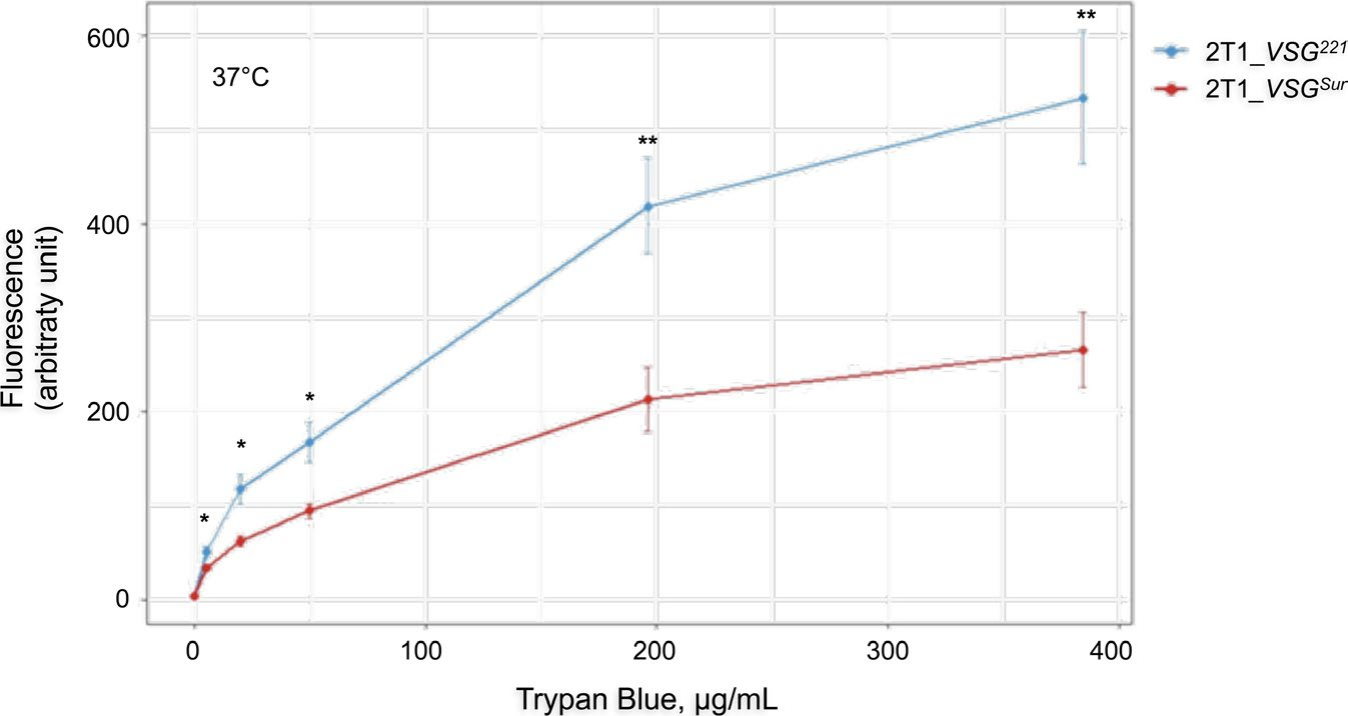
Trypan blue uptake. The trypan blue uptake of *VSG^Sur^* and *VSG^221^* expressing cells as determined by FACS after incubation for 2 h at 37°C. Error bars represent standard deviations of three independent experiments (*P < .05; **P < .01, paired *t* test)

We exploited the fact that trypan blue becomes fluorescent when bound to proteins^18^, ^19^ to investigate drug uptake by *T. b. brucei* 2T1. Cells were incubated with different concentrations of trypan blue at 37°C and subsequently the fluorescence of cell‐associated trypan blue quantified by flow cytometry. In the cell size versus fluorescence scatters, gates were set to exclude dead cells, which give a much stronger signal than live cells.^19^ *VSG^Sur^*‐expressors showed a 30%‐50% reduction in fluorescence when compared with their *VSG^221^* expressing parent (Figure 2). This is consistent with the small increase in the IC_50_ towards this compound.

### 3.3 VSG^Sur^‐mediated suramin resistance is not linked to ISG75

**Figure 3:**
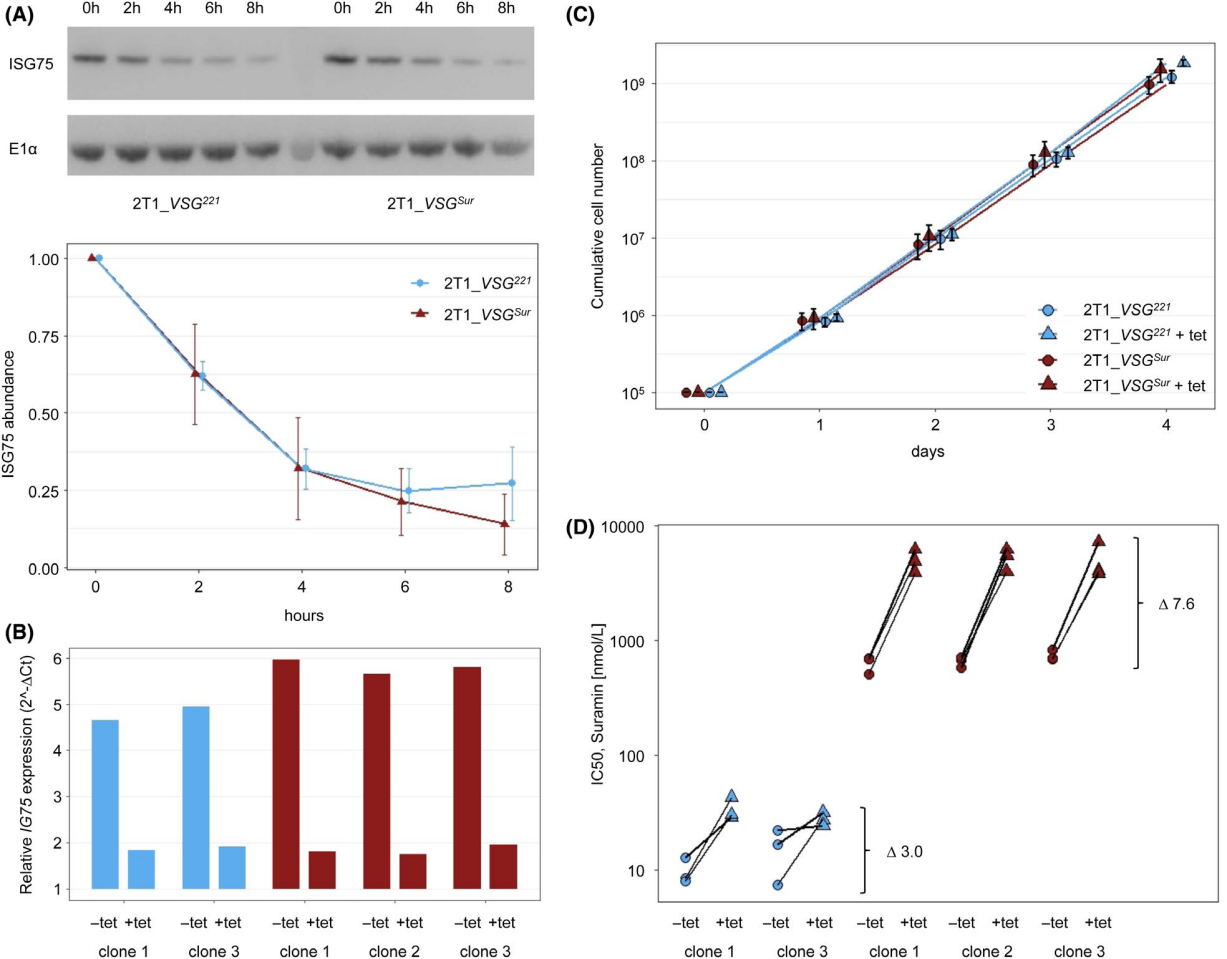
ISG75 expression and effect on suramin sensitivity (blue: parental, *VSG^221^* expressing cells; red: *VSG^Sur^* expressing cells). A, ISG75 turnover as determined by western blot after inhibition of translation for different time spans. Plotted data points represent mean amounts of protein from two independent experiments, for each experiment two technical replicates (western blots) were made. B, Reduction in *ISG75* expression levels upon RNAi knockdown. Gene expression levels are normalized to the housekeeping gene *TERT*, values shown are derived from technical duplicates. C, Cumulative growth curves of *VSG^Sur^* and *VSG^221^* expressing cells ± ISG75 knockdown. Values shown are the averaged growth curves for each group, including two biological replicates from each clone (2T1_wt_c1 and c3 for the *VSG^221^*‐expressor and 2T1_sur_c1, c2, and c3 for the VSG^Sur^‐expressor). The data points represent mean cell numbers plotted with the position_dodge function in R to allow visual distinction between the groups. Error bars represent standard deviation. D, 50% inhibitory concentrations for suramin of *VSG^Sur^* and *VSG^221^* expressing cells ± ISG75 knockdown. ∆ represents the factor of increase upon ISG75 knockdown

Since some suramin resistance can be caused by ISG75 depletion,^6^ we investigated a possible effect of VSG^Sur^ on ISG75 turnover. Therefore, cells were incubated with cycloheximide to block protein synthesis, and the steady‐state ISG75 levels were determined after 0, 2, 4, and 6 hours by Western blotting. The ISG75 half‐life was around 3 hours in both the *VSG^221^* expressing and the *VSG^Sur^* expressing trypanosomes (Figure 3A). We further investigated the effect of ISG75 depletion in *VSG^Sur^*‐expressors (2T1_sur1) and parental cells (2T1_wt) by introduction of a pRPA‐based, tetracycline‐inducible RNAi knock‐down construct. Induction of RNAi for 72 hours led to a 75%‐85% reduction in *ISG75* mRNA levels in 5 (2T1_wt_c1 and c3; 2T1_sur1_c1 to c3) of 6 clones as determined by qPCR (Figure 3B). Growth was slightly faster under knock‐down of *ISG75* with an average population doubling time of 6.9 hours in the presence of tetracycline compared to 7.2 hours without tetracycline (*P*‐value = .003, paired t‐test including all clones, Figure 3C). Suramin sensitivity decreased in all the tested clones upon down‐regulation of ISG75 (Figure 3D). The presence of ISG75‐dependent suramin uptake also in the VSG^Sur^‐expressors indicates that this is not the pathway affected by VSG^Sur^. Moreover, the effect of *ISG75* silencing on suramin sensitivity was clearly stronger (*P* = .009, Welch Two Sample t‐test) in the *VSG^Sur^*‐expressors: the IC_50_ of suramin upon ISG75 knock‐down increased 2.2‐ and 3.8‐fold in parental 2T1 cells, and 7.9‐, 8.1‐, and 6.7‐fold in *VSG^Sur^*‐expressors (Figure 3D). This is in agreement with the above hypothesis, that a pathway distinct from ISG75 is affected and hence, ISG75 confers a larger proportion of suramin uptake in the *VSG^Sur^* expressing trypanosomes.

### 3.4 Effects of serum proteins on suramin susceptibility

**Figure 4:**
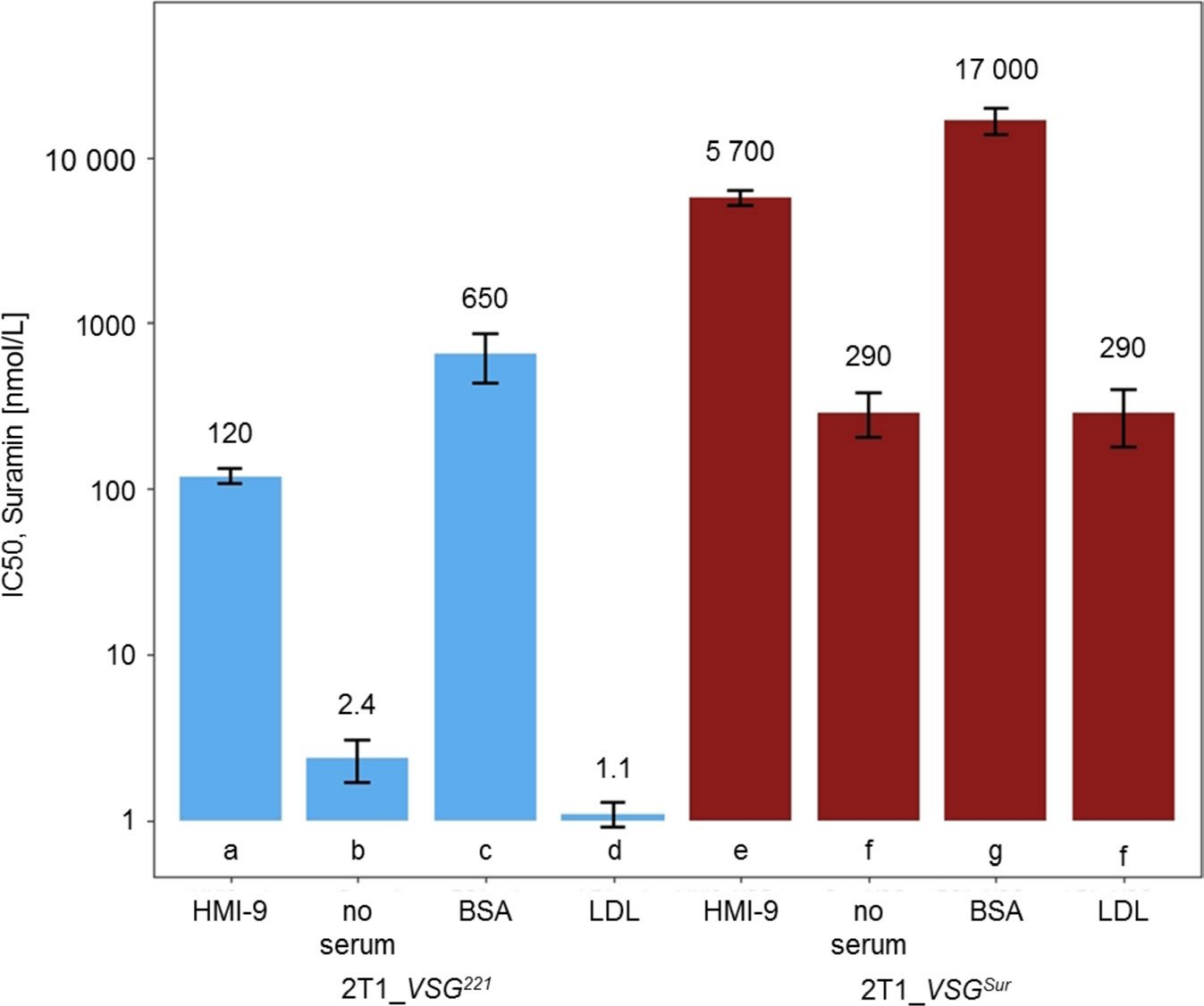
50% inhibitory concentrations in the presence of different serum proteins. Parasites were incubated either in standard growth medium with 15% inactivated horse serum (HMI‐9), in HMI‐medium without any serum (no serum), in HMI‐medium without serum supplemented with albumin (BSA), or in HMI‐medium without serum supplemented with LDL (LDL). Values that differed significantly (*P* < .05, Tukey's multiple comparison test on logarithmic data) are labeled with different letters (a‐g) (independent biological replicates: n = 3 for “HMI‐9” and “BSA”; n = 4 for “no serum,” and “LDL”; each biological replicate consisted of two technical replicates)

Having excluded defects toward ISG75 turnover as a reason for the decreased suramin sensitivity of *VSG^Sur^* expressing cells, we tested for the involvement of serum proteins in VSG^Sur^‐mediated suramin resistance. This had to be performed with wash‐out assays since the trypanosomes cannot be cultivated without serum proteins over long periods. Parental, *VSG^221^* expressing 2T1 cells and *VSG^Sur^*‐expressors were incubated for 4 hours with suramin in HMI‐9 medium without serum proteins, in HMI‐9 supplemented with albumin, with LDL or with serum, respectively, washed, and incubated in standard growth medium (without suramin) for 3 days. As expected, the IC_50_ in these assays (Figure 4) were higher than in the standard 72 hours assay, since suramin was only present for the first 4 hours. Nevertheless, cells incubated in medium without any serum proteins were highly sensitive to suramin: they showed a decrease in IC_50_ of 98% for *VSG^221^* and of 95% for *VSG^Sur^*‐expressing cells when compared with the IC_50_ of cells incubated in medium with serum. This demonstrates that free suramin is taken up by trypanosomes. The addition of bovine serum albumin (BSA) counteracted suramin toxicity, indicating that suramin is sequestered by binding to albumin, which is in agreement with previous findings.^5^ The same effect can also explain why the trypanosomes were less suramin sensitive in the presence of serum than without serum. The addition of LDL further increased suramin susceptibility of serum‐free cultures (50% decrease in IC_50_; Figure 4). However, this effect was only observed in parental, *VSG^221^* expressing cells but not in the *VSG^Sur^* expressing cells (*P*‐value = .008, paired *t *test). These data suggest that *VSG^Sur^* expression abolished LDL‐mediated uptake of suramin.

### 3.5 The effect of VSG^Sur^ and suramin on LDL uptake

**Figure 5:**
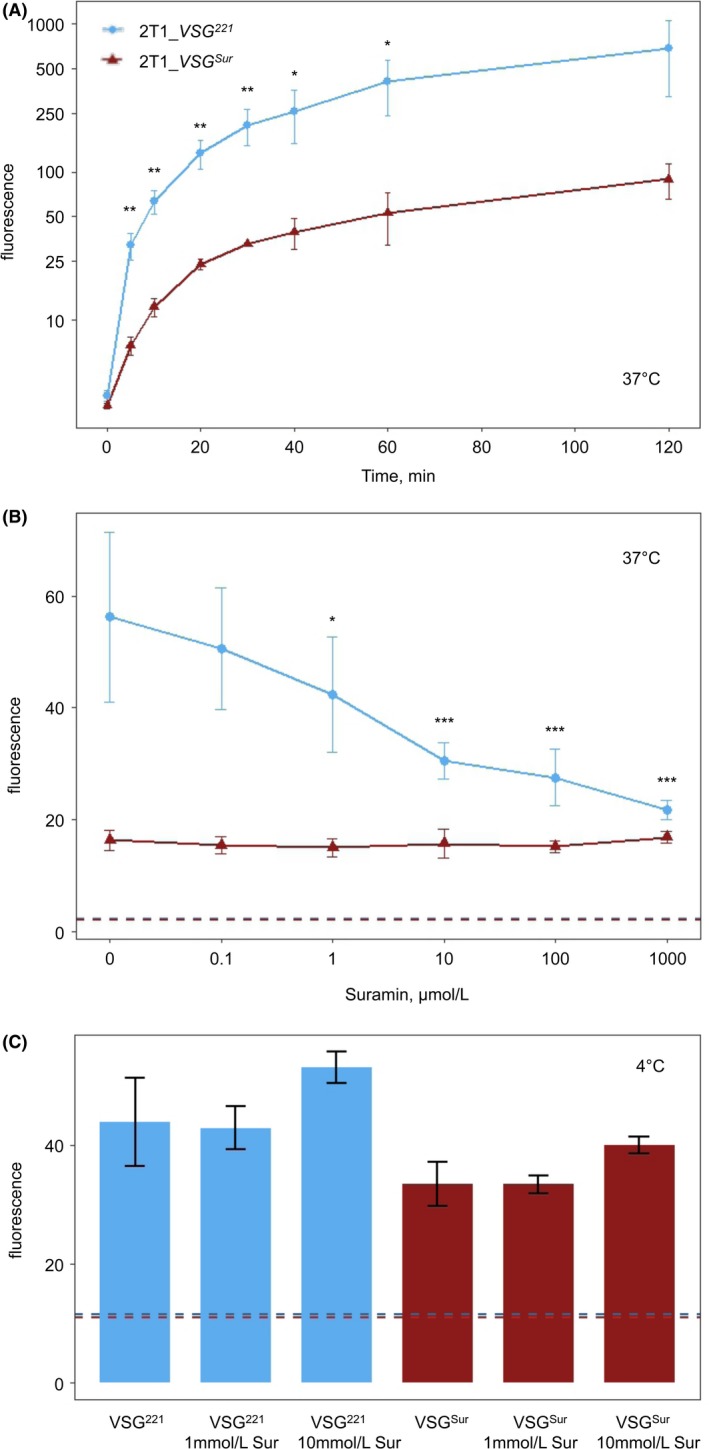
Uptake and binding of fluorescently labeled low‐density lipoprotein and the effect of suramin. A, Uptake of low‐density lipoprotein over a period of 2 h, error bars represent standard deviations (*P < .05; **P < .01; n = 3 for timepoint 120 min; n = 4 for timepoints 0‐60 min; paired *t *test). B, Impact of suramin on the uptake of LDL during 20 min of incubation, asterisk represent significant p‐values if fluorescence of *VSG^221^* expressing cells under incubation with suramin is compared to the fluorescence of *VSG^221^* expressing cells under incubation without suramin (*P < .05; **P < .01; ***P < .001; n = 3, pairwise t‐test with Bonferroni correction), error bars represent standard deviations. Dotted lines represent baseline, which was defined as fluorescence of cells which were incubated with the same amount of LDL but on ice to inhibit endocytosis. C, Binding of LDL to the cell surface. Dotted lines represent background fluorescence of *VSG^221^* expressing (blue) and *VSG^Sur^* expressing (red) cells incubated without fluorescently labeled LDL. Error bars represent standard deviations of three measurements (two for 10 mmol/L suramin)

To measure uptake of LDL, *T. b. brucei* 2T1 cells were incubated for different times with fluorescently labeled LDL at 37°C to allow endocytosis, and the internalized LDL was quantified using FACS. No plateau was reached within 120 minutes incubation time, neither for *VSG^221^* nor for *VSG^Sur^* expressing cells (Figure 5A). LDL uptake was highly reduced in *VSG^Sur^* expressing cells with an 80%‐90% lower fluorescence than *VSG^221^*‐expressors throughout the 5 to 120 minutes period (Figure 5A). The presence of suramin led to reduction in LDL uptake in wild‐type cells but not in *VSG^Sur^*‐expressors (Figure 5B). To differentiate whether the reduced LDL uptake is mediated through a reduced binding or endocytosis, we looked at the binding of LDL to the cell surface. LDL binding at 4°C was around 30% reduced in *VSG^Sur^* compared to *VSG^221^* expressing cells (overall *P*‐value = .0003, paired t‐test). Thus, the highly reduced LDL uptake cannot be explained by a reduction in the binding alone and a reduced endocytosis seems to play a major role. No effect on binding was observed in the presence of suramin (Figure 5C).

### 3.6 The effect of VSG^Sur^ and suramin on transferrin uptake

**Figure 6:**
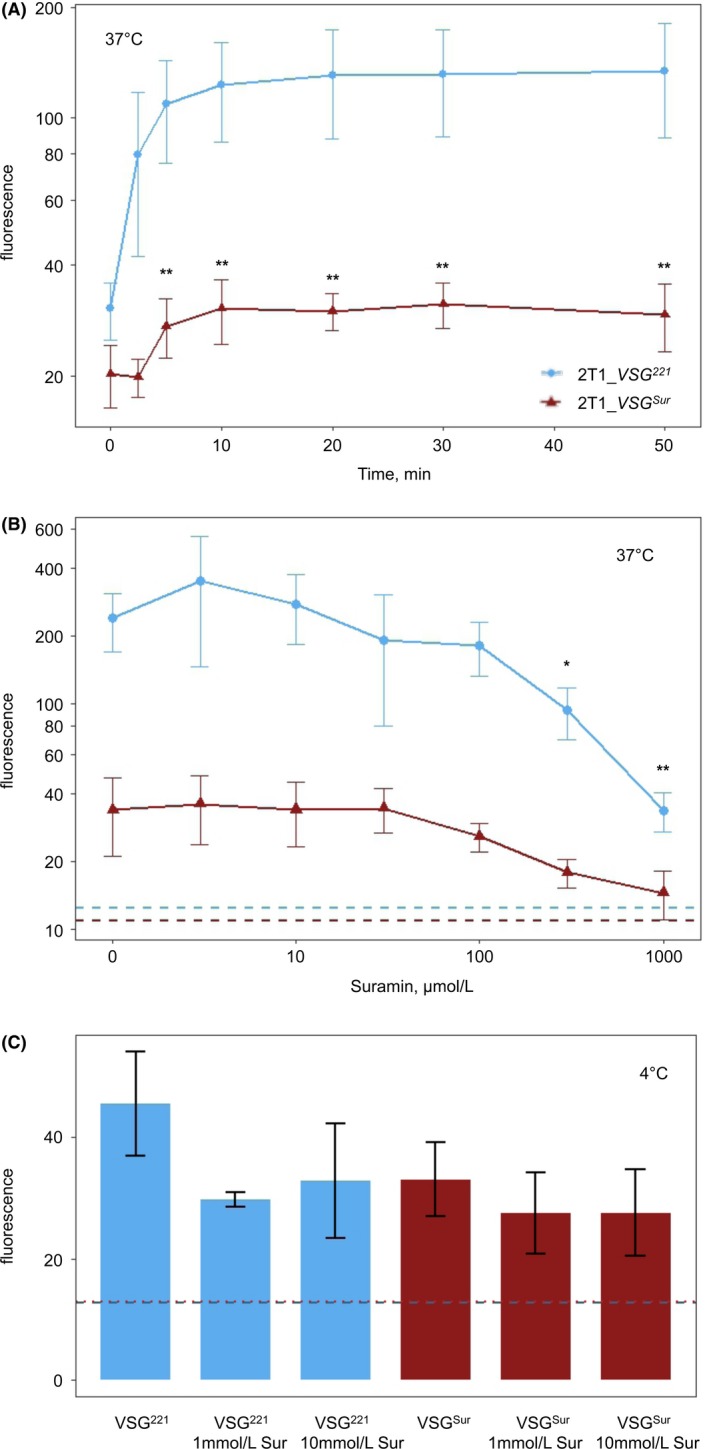
Uptake and binding of fluorescently labeled transferrin and the effect of suramin. A, Transferrin uptake of *VSG^Sur^* and *VSG^221^* expressing cells at 37°C over time. Error bars represent standard deviation of four independent experiments (*P < .05; **P < .01, paired *t *test). B, Influence of suramin on transferrin uptake. Data of three independent experiments are shown, error bars represent standard deviations. Dotted lines represent baseline, which was defined as fluorescence of cells which were incubated with the same amount of transferrin but on ice to inhibit endocytosis. C, Transferrin binding to the cell surface with and without suramin as determined after incubation of cells on ice. Dotted lines represent background fluorescence of *VSG^221^* expressing (blue) and *VSG^Sur^* expressing (red) cells incubated without fluorescently labeled transferrin. Error bars represent standard deviations of three measurements (two for 10 mmol/L suramin)

We investigated uptake of transferrin as an additional substrate that is imported by receptor‐mediated endocytosis.^20^, ^21^ Again, trypanosomes were incubated for different periods with fluorescently labeled transferrin at 37°C and analyzed by FACS. In *VSG^221^* expressing 2T1 cells transferrin was quickly internalized, reaching a plateau of more than four times background fluorescence after ten minutes (Figure 6A). In contrast, *VSG^Sur^* expressing 2T1 cells incubated with labeled transferrin only showed a slight increase in fluorescence to 1.5 times background level (Figure 6A), which corresponds to an 80% lower fluorescence than observed in parental cells. Since transferrin uptake had been described to be reduced in the presence of suramin in HeLa cells,^22^ we investigated the effect of suramin on transferrin uptake in *T. brucei*. Cells were incubated with fluorescently labeled transferrin in different concentrations of suramin for 10 minutes (the incubation time was kept short to limit the toxic effects of suramin). At concentrations below 300 µmol/L, suramin had no effect on transferrin uptake; only at very high suramin concentrations, reduced amounts of intracellular transferrin were measured in *VSG^221^* expressing cells (Figure 6B, *P*‐values of .04 and .006 for concentrations of 300 and 1000, respectively, pairwise *t *test with Benjamini Hochberg correction). To differentiate whether the reduced internalization of transferrin in *VSG^Sur^* expressing cells was caused by reduced binding or reduced endocytosis, we quantified surface binding of transferrin to trypanosomes by incubating the cells on ice. The fluorescence of *VSG^Sur^* expressing cells incubated without suramin was around 40% lower than the fluorescence of the *VSG^221^* expressing cells (Figure 6). Thus again, the highly reduced transferrin uptake cannot be attributed to reduced binding. Upon addition of suramin at 1 mmol/L and 10 mmol/L, the difference between *VSG^221^* and *VSG^Sur^*‐expressors became smaller (Figure 6C). However, at such high suramin concentrations we cannot exclude an ionic effect. The overall p‐value of the difference between *VSG^221^* and *VSG^Sur^*‐expressors was 0.03.

### 3.7 The effect of VSG^Sur^ on VSG endocytosis

**Figure 7:**
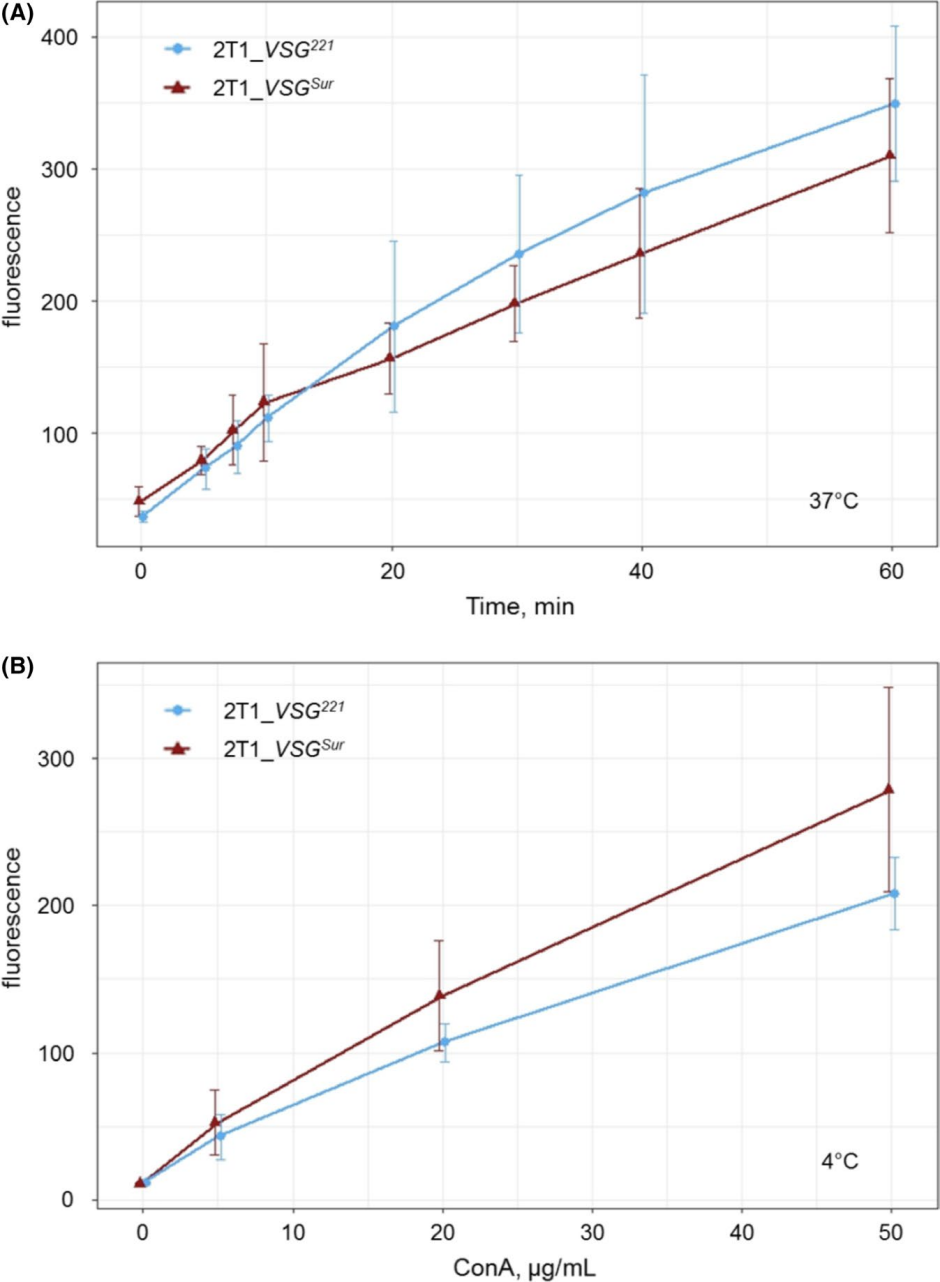
Concanavalin A uptake and binding in *VSG^Sur^* and *VSG^221^* expressing cells. A, Fluorescence of cells incubated with 5 µg/mL labeled ConA at 37°C, time point zero represents cells that were kept on ice before the addition of ConA in order to allow binding but not uptake. Error bars represent standard deviations of five (for timepoint 60 only four) independent experiments. B, Fluorescence of surface bound ConA of cells incubated with different ConA concentrations at 4°C to prevent endocytosis. Error bars represent standard deviation of four independent experiments

Variant surface glycoproteins are endocytosed at a very high rate with a turnover equivalent to the whole surface coat within 12 minutes.^23^ To test whether *VSG^Sur^* expression has a general impact on endocytosis, we investigated endocytosis of *VSG^Sur^* and *VSG^221^*. Concanavalin A (ConA) is a lectin that binds to glycosylated surface proteins, in case of trypanosomes mainly to VSG.^24^ It is endocytosed together with the VSG and can therefore be used to measure VSG endocytosis rates. Cells were incubated for time periods of 0 to 60 minutes at 37°C with 5 µg/mL fluorescently labeled ConA. Subsequently cells were fixed, and the fluorescence of cell‐associated ConA was measured by flow cytometry. Up to an incubation period of 20 minutes fluorescence levels were in the same range or even higher for *VSG^Sur^* than for *VSG^221^*‐expressors. Thereafter *VSG^Sur^*‐expressors showed a slightly lower fluorescence than *VSG^221^*‐expressors (Figure 7A). Since ConA does not bind equally well to all VSGs^24^ we examined the ConA‐binding capacity of the two cell lines. The cells were incubated with different concentrations of ConA on ice to prevent endocytosis, and surface bound ConA was quantified by flow cytometry. *VSG^Sur^* expressing cells showed a 22%‐34% higher fluorescence than *VSG^221^*‐expressors (*P*‐value = .0097, paired t‐test, Figure 7B). Taken together, the facts that *VSG^Sur^*‐expressors showed similar levels of internalized ConA as *VSG^221^*‐expressors at 37°C but more surface bound ConA at 4°C, indicate that endocytosis of VSG may be slightly reduced in *VSG^Sur^* expressing cells. However, we observed no enlargement of the flagellar pocket, which is a hallmark for blocked endocytosis.

### 3.8 The effect of VSG^Sur^ on sensitivity to human serum

**Figure 8:**
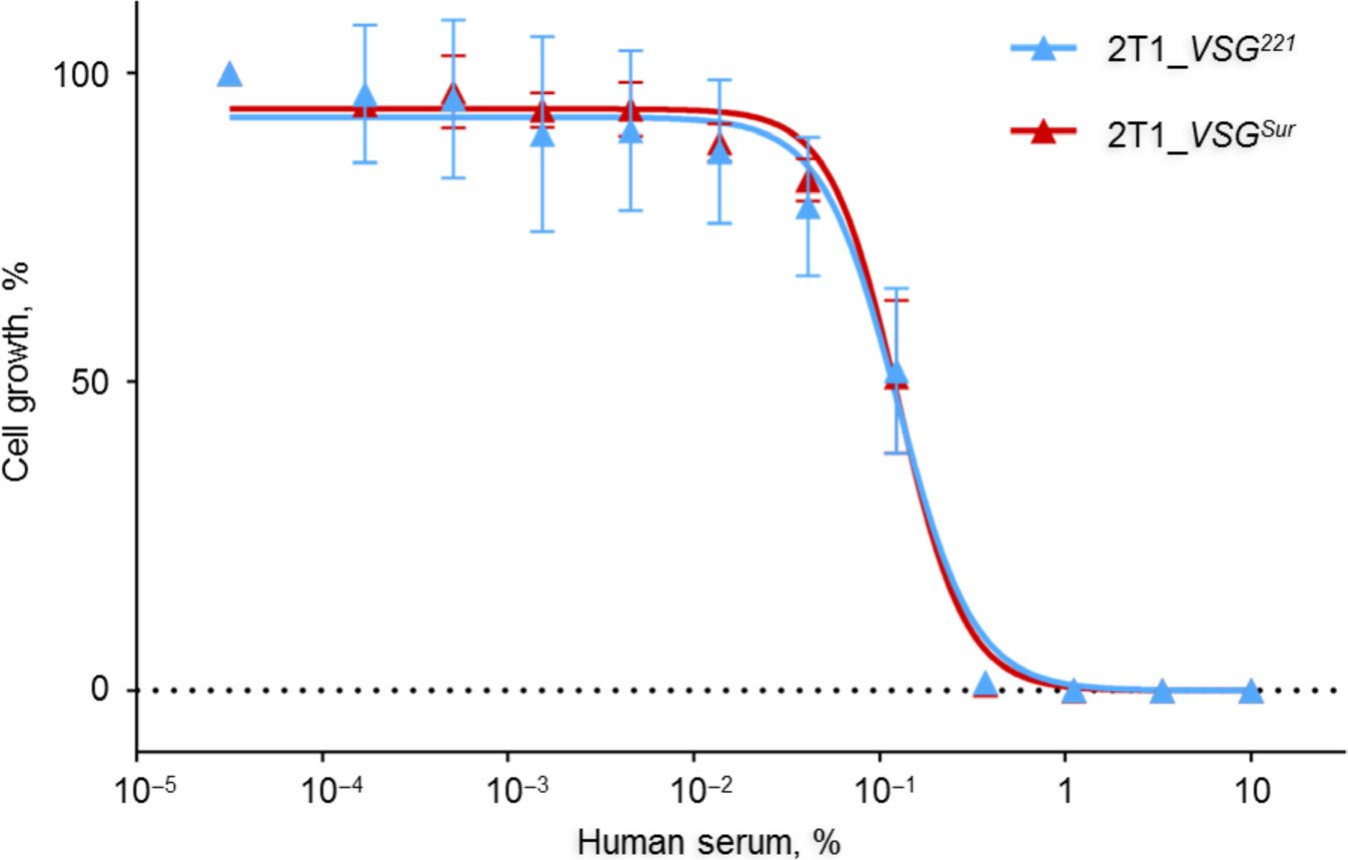
Sensitivity to normal human serum

Most trypanosome species other than *T. b. rhodesiense* and *T. b. gambiense* are lysed by the two trypanolytic factors (TLF1 and TLF2) present in human serum. TLF1, a high‐density lipoprotein complex, and TLF2, a protein complex, both contain apolipoprotein L1,^25^ which mediates trypanolysis.^26^ TLF1 is much more active than TLF2 and kills *T. b. brucei* at very low concentrations in the absence of competing haptoprotein.^27^ TLF1 binds to the haptoglobin‐hemoglobin receptor (TbHpHbR) in the flagellar pocket and is subsequently endocytosed.^28^ To test whether the endocytosis of the TbHpHbR is affected by *VSG^Sur^* expression, sensitivity to human serum was tested. However, bloodstream‐form *T. b. brucei* 2T1 expressing *VSG^Sur^* or *VSG^221^* were equally sensitive (Figure 8). This demonstrates that endocytosis of the trypanolytic factor of human serum, and the downstream mechanisms of cell lysis, are not affected by VSG^Sur^.

## 4 DISCUSSION

We have originally identified VSG^Sur^ as the VSG expressed in bloodstream‐form *T. brucei* spp. that had been selected in vitro for resistance to suramin.^11^ The resistant cells had IC_50_ values to suramin of around 1.2 µmol/L, which was almost 100‐fold above the IC_50_ of the parental cells but still below the suramin plasma levels of 70 µmol/L in treated patients.^29^ Thus the expression of VSG^Sur^ is unlikely to cause suramin treatment failure in patients. Nevertheless, it is intriguing how a particular VSG can be linked to drug susceptibility.

Here we show that transgenic expression of *VSG^Sur^* is sufficient to cause suramin resistance in *T. b. brucei*, which is otherwise highly sensitive to the drug. Based on the reduced uptake of trypan blue by the cells that express *VSG^Sur^*, our working hypothesis is that suramin resistance is mediated by a reduced drug uptake. Trypanosomes take up suramin through receptor‐mediated endocytosis.^4^, ^5^ Endocytosis in trypanosomes takes place at the flagellar pocket and the most abundantly endocytosed protein is VSG itself. VSGs are endocytosed into Rab5a endosomes^30^ at a very high rate.^23^ Subsequently the VSG is recycled back to the trypanosome surface by exocytosis via Rab11 exocytic carriers.^31^

Concanavalin A, a lectin which binds to VSG, can be used as a marker to track VSG internalization.^32^ We did not observe distinct alterations of ConA endocytosis in *VSG^Sur^* expressing trypanosomes, neither by flow cytometry nor by fluorescence microscopy (not shown) and conclude that if there is an effect on VSG internalization at all, it is only marginal. Transferrin and low‐density lipoprotein (LDL) are important nutrients for African trypanosomes.^33^, ^34^ Transferrin binds to the transferrin receptor, which is a heterodimer of ESAG6 and ESAG7, and is subsequently endocytosed in Rab5a endosomes similar to the VSGs.^30^ Once the ligand is removed, transferrin receptors are recycled to the cell membrane. Similar to VSG, this happens at a very high rate with a turnover of only 11 minutes.^35^ LDL is imported via receptor‐mediated endocytosis as well. The LDL receptor is supposedly located at the flagellar pocket and on the flagellar membrane,^36^ but it has not been identified. As determined by flow cytometry, the *VSG^Sur^*‐expressors showed highly reduced intracellular levels of fluorescently labeled transferrin and fluorescently labeled LDL after incubation at 37°C. This suggests that *VSG^Sur^* expression impairs endocytosis of the LDL receptor and the transferrin receptor. However, the expression of *VSG^Sur^* did not alter sensitivity to human serum, indicating that endocytosis of the TbHpHb receptor is not affected. The HpHb receptor is a glycosylphosphatidylinositol (GPI) anchored, glycosylated surface protein located at the flagellar pocket.^28^ Taken together, *VSG^Sur^*‐expressors showed a normal growth and no enlarged flagellar pockets, thus we assume that VSG^Sur^ has no effect on bulk endocytosis. Receptor‐mediated endocytosis is affected by VSG^Sur^, but only specific pathways. While endocytosis of transferrin is highly reduced, endocytosis of the VSG may be moderately reduced, and endocytosis of the HpHb receptor unaffected by *VSG^Sur^* expression. Additionally, and most importantly for suramin susceptibility, endocytosis of the elusive LDL receptor is highly reduced.

Suramin inhibited uptake of LDL by *VSG^221^* expressing cells, in agreement with published results,^5^ but not in *VSG^Sur^* expressing cells. Short‐term suramin wash‐out experiments in serum‐free medium indicated that suramin uptake is lowest in the presence of albumin and intermediate in the presence of serum, which confirms the literature.^5^ We further show for the first time that suramin is also taken up in the absence of serum. The addition of LDL appeared to increase suramin uptake in *VSG^221^* expressing cells, which is in accordance with the literature,^5^ but not in *VSG^Sur^*‐expressors. On the other hand, knockdown of ISG75 had a stronger effect on suramin sensitivity in *VSG^Sur^*‐expressors than in *VSG^221^* expressing cells.

Suramin remains an enigmatic molecule with polypharmacology and multiple potential uses. The effects of suramin on vesicular trafficking are likely to be complex, since suramin can interfere with vesicular transport and is imported via receptor‐mediated endocytosis itself.^4^, ^5^, ^7^ Our findings support a model of two independent pathways for endocytosis of suramin by trypanosomes: *via* the invariant surface glycoprotein ISG75 that directly binds suramin, and *via* the LDL receptor, a protein of unknown nature that binds suramin complexed to LDL. While the former is still active in cells expressing *VSG^Sur^*, the latter is strongly impaired, causing suramin resistance in *T. brucei*.

## CONFLICT OF INTEREST

The authors have declared that no competing interests exist.

## AUTHOR CONTRIBUTIONS

N. Wiedemar, M. Zoltner, M. C. Field, and P. Mäser designed the research. N. Wiedemar, M. Zwyer, M. Zoltner, and M. Cal performed the research. N. Wiedemar, M. Zwyer, M. Zoltner, M. C. Field, and P. Mäser analyzed data. N. Wiedemar and P. Mäser wrote the paper.
